# Disseminated Tuberculosis Masquerading as a Uterine Cervical Mass in an Immunocompetent Young Female: A Case Report

**DOI:** 10.7759/cureus.70883

**Published:** 2024-10-05

**Authors:** Sagar Varma, Lakshmi M, Swathy Moorthy, Emmanuel Bhaskar

**Affiliations:** 1 Internal Medicine, Sri Ramachandra Medical College and Research Institute, Sri Ramachandra Institute of Higher Education and Research, Chennai, IND

**Keywords:** anti-tuberculosis drugs, disseminated tuberculosis, genito-urinary tuberculosis, infectious disease medicine, renal tuberculosis

## Abstract

Concurrent tubercular involvement of two or more non-contiguous organs is termed disseminated tuberculosis (TB) and is rare in immunocompetent patients. We describe the case of a young immunocompetent woman with disseminated TB who presented with primary complaints of amenorrhea and dysuria. Abdominal ultrasound showed a uterine cervical mass, which on histopathological evaluation revealed epithelioid granulomata with Langhans giant cells and acid-fast bacilli (AFB). Her chest radiograph showed scattered air space opacities bilaterally, and contrast-enhanced computed tomography of the abdomen revealed the involvement of bilateral kidneys, para-aortic lymph nodes, adrenals, sacroiliac regions, and the gastrointestinal tract. A colonoscopy picked up an ulcer in the terminal ileum, which on histopathology was positive for AFB. The patient was started on anti-tubercular treatment.

## Introduction

Pulmonary tuberculosis (TB) is very common in developing countries like India, accounting for high morbidity and mortality. India accounted for 28% of all estimated incident TB cases worldwide in 2021 [1]. The exact incidence of disseminated TB globally is still unclear; however, among immunocompetent adults, disseminated TB is thought to comprise <2% of all TB cases and up to 20% of all extrapulmonary TB cases. Subtle and nonspecific clinical presentations of disseminated TB are barriers to accurate diagnosis, implying that the incidence is vastly underestimated [2]. Disseminated TB is generally diagnosed among immunosuppressed individuals and is rare in immunocompetent patients. The definitive diagnosis relies on histopathological and microbiological findings. Here, we describe a young lady with disseminated TB presenting with secondary amenorrhea and manifesting as a uterine cervical mass on ultrasonography (USG).

## Case presentation

A female in her late 20s came to us with amenorrhea for four years, as well as painful micturition for four months, associated with urge incontinence and hematuria for one month. She also noticed a low-grade fever for one month, associated with an evening rise in temperature. She had shortness of breath on exertion for one month, not associated with cough or expectoration, and experienced a loss of appetite, with an 8 kg weight loss over the last three months. She had no history of leakage of urine while coughing, laughing, or straining; continuous dribbling of urine; or decrease in urine output. She did not report spotting, sexual discomfort, or post-coital bleeding. She had a history of one elective lower segment cesarean section, one normal vaginal delivery, and no abortions; both conceptions were spontaneous, and the last childbirth was six years ago, following which she resumed regular periods for about 1.5 years prior to the onset of her current symptoms. No significant family or contact history was reported.

On clinical examination, she had a tired affect. Blood pressure was not recordable on arrival and improved to 100/60 mmHg after 500 mL of 0.9% normal saline was given as a bolus. She was pale and had bilateral pitting pedal edema. No lymphadenopathy was noted. Chest auscultation revealed bilateral diffuse crepitations. Abdominal examination revealed a Pfannenstiel scar and was non-tender and soft in consistency, with no palpable organomegaly or free fluid. On per-speculum examination, a 2 x 3 cm polypoidal mass was seen through the uterine cervix, with no ulceration, friability, or bleeding on touch. Cardiovascular and neurological examinations were normal. Laboratory parameters are as follows, as shown in Table 1.

**Table 1:** Laboratory parameters at admission ESR, erythrocyte sedimentation rate; BUN, blood urea nitrogen; AST, aspartate aminotransferase; ALT, alanine aminotransferase; hpf, high power field

Parameter	Patient value	Reference range
Hemoglobin (g/dL)	8	12-15
Total white cell counts (cells/μL)	5,260	4,000-11,000
Platelet count (10^3^ cells/μL)	337	150-450
ESR (mm/hr)	21	4-12
Serum ferritin (μg/L)	253.70	13-150
BUN (mg/dL)	11	7-18
Serum creatinine (mg/dL)	0.9	0.6-1.3
AST (IU/L)	22	<35
ALT (IU/L)	9	<35
Serum albumin (g/dL)	2.1	3.9-4.9
Serum globulin (g/dL)	2.7	2-3.5
Urine protein by dipstick	4+	Nil
Urine leukocyte esterase by dipstick	3+	Nil
Urine pus cells by microscopy (per hpf)	Plenty	3-5
Urine casts by microscopy	Nil	Nil
24-hour urine protein collection (mg)	582	<300
Urine culture	No growth	-
Serum 8 AM cortisol (μg/dL)	17.45	6-19

**Figure 1:**
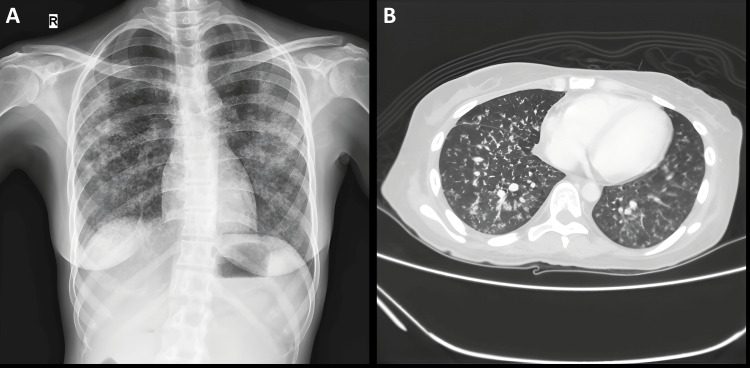
Initial chest imaging Pane (A): Chest X-ray showing bilateral scattered air space opacities. Pane (B): CT thorax showing multiple centrilobular nodules in bilateral lower lung fields. CT, computed tomography

Chest imaging with X-ray and computed tomography (CT) is shown as follows in Figure 1.

**Figure 2:**
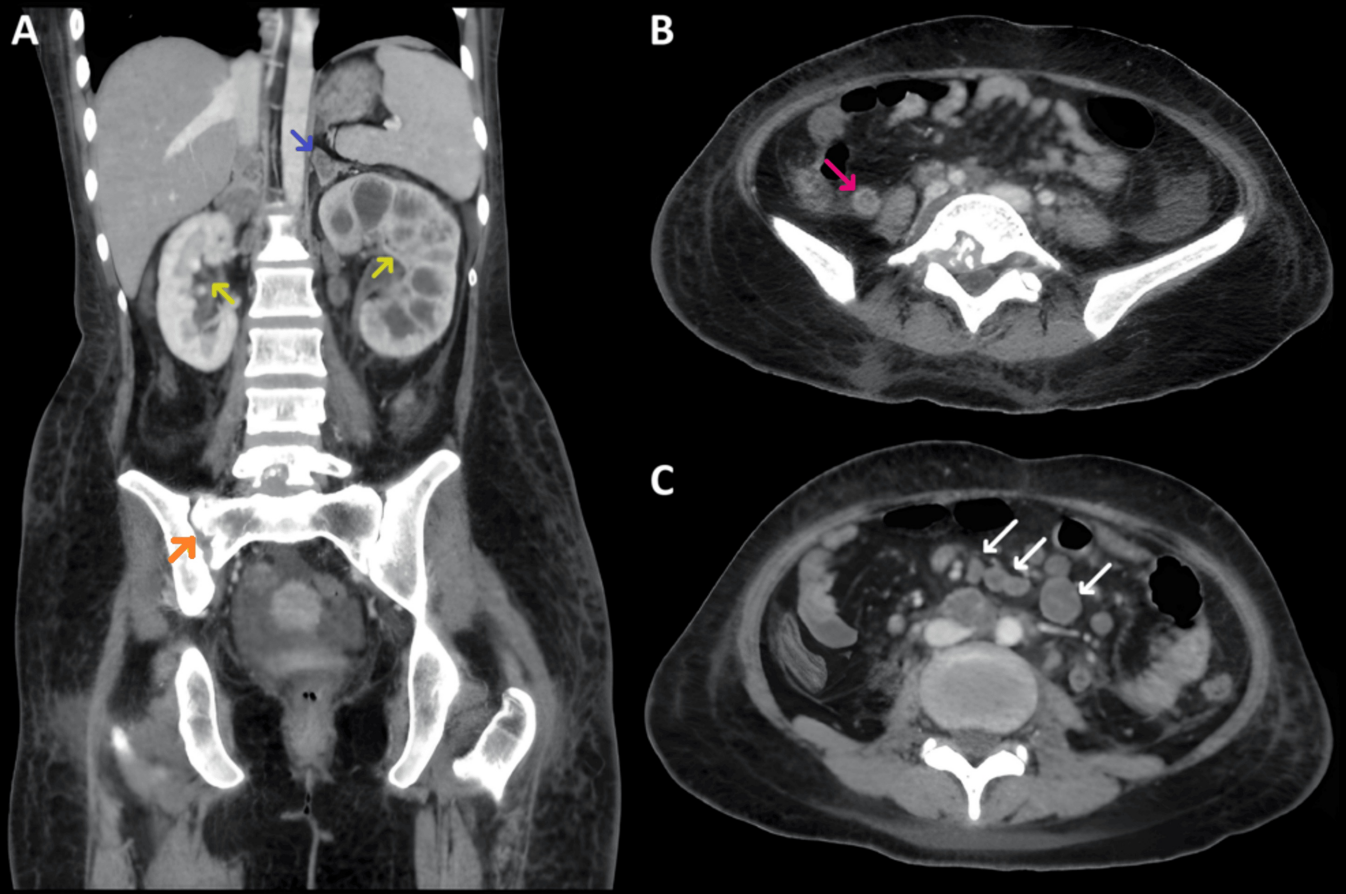
CECT of the abdomen Pane (A): Bilateral bulky adrenal glands with large conglomerate, peripherally enhancing, hypodense lesions within (blue arrow); bulky left kidney with moderate hydronephrosis (green arrows); and infective right sacroiliitis with L5 lytic lesion (orange arrow). Pane (B): Mild thickening of the ileocecal junction and cecum, with a secondarily inflamed appendix (pink arrow). Pane (C): Necrotic abdominal lymphadenopathy (white arrows). CECT, contrast-enhanced computed tomography

USG of the abdomen showed a solid polypoidal mass of 2.6 x 1.8 cm at the external uterine cervical os, with the base within the cervical canal measuring 1.4 x 0.5 cm, along with bilateral hydroureteronephrosis and multiple intrabdominal nodal enlargements. The uterine corpus, fallopian tubes, and ovaries were noted to be normal. Contrast-enhanced CT (CECT) of the abdomen showed features suggestive of TB (Figure 2).

**Figure 3:**
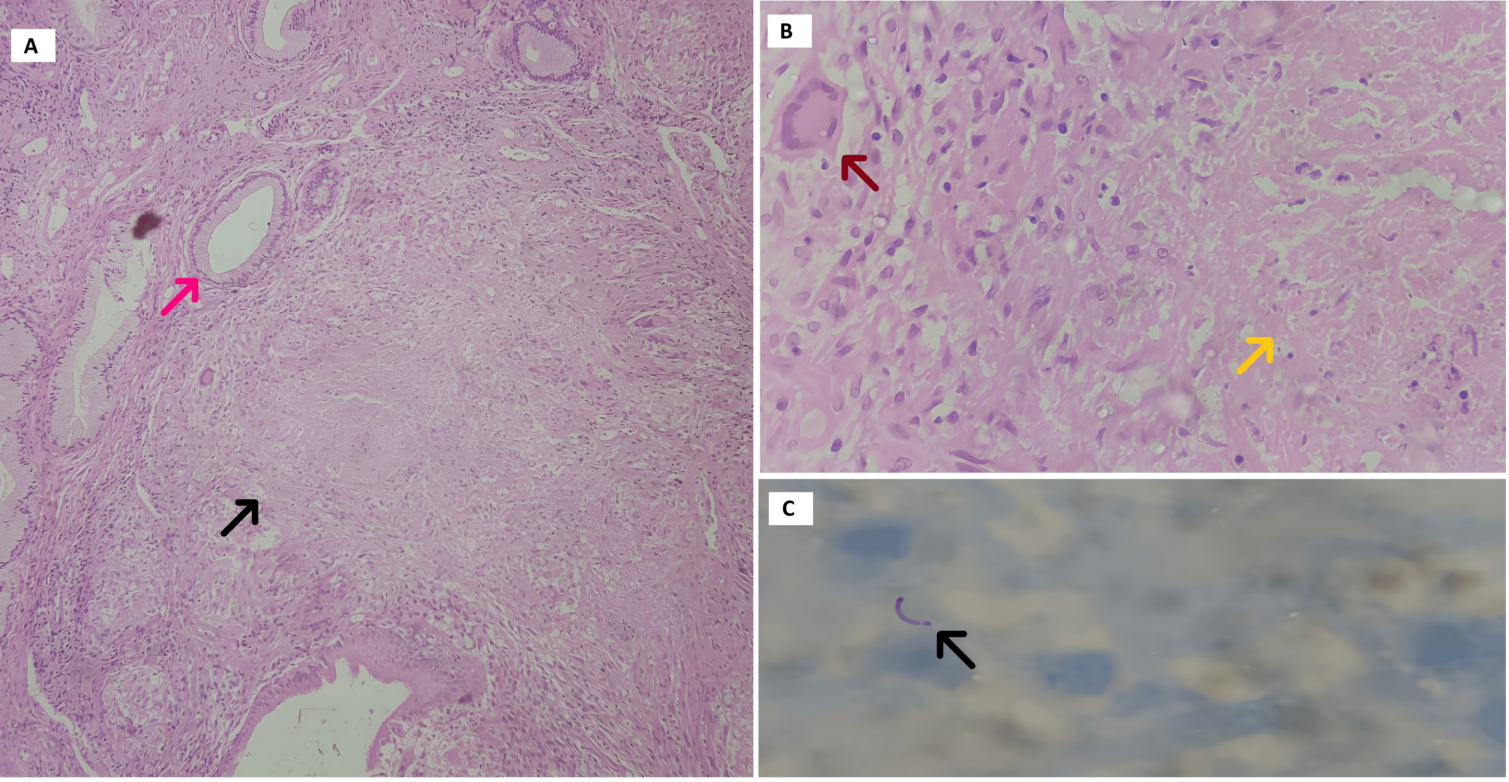
Histopathological examination of the cervical punch biopsy specimen Pane (A): Histopathological microphotograph showing the presence of granuloma (black arrow) with surrounding endocervical glands (pink arrow) (10x magnification). Pane (B): Granuloma composed of central caseous necrosis (orange arrow), few epithelioid histiocytes and Langhans multinucleated giant cell (brown arrow) (40x magnification). Pane (C): AFB stain showing acid-fast bacilli (black arrow) (100x magnification). AFB, acid-fast bacilli

Colonoscopy showed the presence of terminal ileal ulcers, biopsies of which showed multiple well-defined epithelioid granulomas with Langhans multinucleated giant cells, cryptitis, and acute inflammatory granulation tissue. Areas of necrosis in the ileocecal valve, caecum, and numerous clusters of acid-fast bacilli (AFB) with a high bacillary load were also noted. As no contrast excretion was seen in the left kidney in the delayed phases, a technetium-99 m-labeled diethylene triamine penta-acetic acid (DTPA) scan was done, which showed that the left kidney was underperfused and hydronephrotic, with poor parenchymal tracer uptake (glomerular filtration rate, or GFR 12.1 mL/min), while the right kidney was normal-sized and well-perfused (GFR 62.5 mL/min). Sputum smear microscopy with AFB stain and cartridge-based nucleic acid amplification testing (CB-NAAT) were negative for *Mycobacterium tuberculosis*. Under the suspicion of cervical carcinoma, a Pap smear was taken, which showed only an inflammatory picture, and a cervical punch biopsy was performed, showing fragments of endocervical tissue with multiple well-defined epithelioid granulomas, Langhans giant cells, and necrosis, positive for AFB (Figure 3). Thus, the patient was determined to have pulmonary, abdominal, adrenal, vertebral, and genitourinary TB.

The patient was started on weight-based antitubercular therapy (ATT) with isoniazid, rifampicin, pyrazinamide, and ethambutol for two months, followed by isoniazid and rifampicin for four months. Following the initiation of ATT, the patient gained 3 kg of weight. Since discharge, the patient has been followed up periodically and has further gained 5 kg of weight, with resumption of her menses and resolution of urinary symptoms.

## Discussion

Disseminated TB

Disseminated TB can occur secondary to the progression of a primary TB focus or the reactivation of a latent focus, with subsequent lymphohematogenous spread [3]. One proposed mechanism is pulmonary TB leading to alveolar epithelial layer erosion and the spread of infection to the pulmonary vein, from where it enters the systemic circulation via the left side of the heart, leading to disseminated TB. Another proposed mechanism is the involvement of lymphatics draining into systemic venous blood that circulates back to the lungs, leading to pulmonary disseminated TB with a miliary appearance [3,4].

Disseminated TB may lead to the involvement of many organs, such as the lung, liver, spleen, bone marrow, adrenals, eyes, thyroid, gastrointestinal tract, and genitourinary tract. Genitourinary tract TB accounts for 27% of extrapulmonary TB worldwide (18% in India), with genital TB alone leading to 9% of all extrapulmonary TB cases [5].

Adrenal TB

The adrenal gland is the most common endocrine organ involved in extrapulmonary TB (up to 6% of cases of active pulmonary TB at autopsy, usually bilateral). Involvement of more than 90% of the gland may lead to a life-threatening Addisonian crisis. On the CECT abdomen, peripheral rim enhancement is seen in half of the affected patients. Mass-like enlargement is observed in around 50-60% of cases, and adreniform hyperplasia is noted in approximately 30-50% of cases [6].

Renal TB

Renal TB is a common manifestation of genitourinary TB, occurring in up to 10% of cases with active pulmonary TB. The pathogenesis involves the lodging of mycobacteria in periglomerular capillaries, forming microscopic granulomas that later evolve into macroscopic granulomas. These can caseate and cavitate, leading to the destruction of the renal parenchyma, ulcerocavernous lesions of the pelvicalyceal system, and rupture or necrosis of papillae, causing sloughing and extension into the collecting system [7].

Imaging plays a crucial role in diagnosing renal TB. X-ray detects characteristic calcifications, including various patterns such as amorphous, curvilinear, and lobar calcifications. Lobar calcification is pathognomonic of TB and is associated with autonephrectomy [8]. USG may reveal granulomata, masses, cavities, uneven caliectasis, and hydronephrosis. CT helps assess severity and identifies calcifications, scars, nodules, cavities, papillary necrosis, and abscesses [9]. Pelvicalyceal involvement presents as ulceration, wall thickening, and strictures. CT also identifies unique patterns, like the "daisy flower" appearance [10]. However, it may not visualize very early TB changes, for which intravenous urography is still the gold standard.

A high clinical suspicion, combined with appropriate imaging techniques, is crucial for the early detection and management of renal TB. In severe cases, nephrectomy may be necessary to remove dormant bacilli. Prompt diagnosis and intervention are essential in preventing associated complications.

Genital TB

The frequency of organ involvement in genital TB in females is as follows: fallopian tubes (95-100%), uterine endometrium (50-60%), ovaries (20-30%), cervix (5-15%), uterine myometrium (2.5%), and vagina/vulva (1%). In males, the epididymis and prostate are most commonly involved. Testicular involvement may occur following direct extension from the epididymis [11]. Most female patients are diagnosed with genital TB during an infertility evaluation. Women in the reproductive age group (15-45 years) are most affected. The majority of cases are asymptomatic or present with infertility. Others may report abnormal uterine bleeding, painful menses, pelvic pain, and abnormal vaginal discharge [12]. Fallopian tube involvement is typically bilateral, with the ampulla and fimbriae commonly involved; the isthmus and interstitial portion may remain uninvolved. Hysterosalpingography is contraindicated due to the risk of exacerbation of subclinical tubal TB and peritonitis. If performed, it will show fallopian tubes with ragged outlines and multiple strictures leading to a beaded appearance. USG may show thickened walls with hydrosalpinx or the presence of debris suggesting pyosalpinx. It may also show loculated ascites, tubo-ovarian masses, and uterine involvement. Uterine involvement is generally localized to the endometrium, probably as a result of cyclic menstrual shedding. Initial presentation may be as ulcerative, granular, or fungating lesions, which may later lead to intrauterine synechiae causing obliteration of the uterine cavity (Asherman syndrome). Ovarian TB is also frequently bilateral, often associated with adhesions and tubo-ovarian masses [6].

Cervical TB

Cervical TB is an uncommon manifestation (0.1-0.65% of all cases of TB) [13] and often grossly develops into ulcerative, polypoidal, miliary, or interstitial patterns. The ulcerative pattern shows a single lesion with well-defined edges that bleeds easily upon touch. Papillary lesions closely resemble miliary carcinoma: the entire cervix is enlarged, with small miliary tubercles visible on the surface. The interstitial type appears initially as a nodule, which progresses to become a necrotic lesion. This, in turn, may be discharged, leaving a cavity behind [14]. Hysterosalpingography may reveal cervical distortions with an irregular endocervical canal and feathery diverticular outpouchings. Since uterine cervical TB can mimic cervical carcinoma, tissue sampling is necessary [6]. In our case, tissue sampling from the cervical mass demonstrated AFB, clinching the diagnosis. Similar cases of secondary amenorrhea as the first presentation of disseminated TB have been described in the literature [15-17].

## Conclusions

Amenorrhea is an uncommon primary manifestation of genitourinary TB, which is usually asymptomatic or presents as infertility in an undiagnosed case of TB. Cervical TB is uncommon, and its presentation as an ulcerative or polypoidal lesion shares a differential with cervical carcinoma. A biopsy in such cases helps secure the diagnosis. Although disseminated TB is more common in immunocompromised cases, it can infect immunocompetent patients and should be thoroughly evaluated. In sputum-negative patients with a suspicion of TB, imaging is of paramount importance to clinch the diagnosis.
